# Two-State or Non-Two-State? An Excess Spectroscopy-based Approach to Differentiate the Existing Forms of Molecules in Liquids Mixtures

**DOI:** 10.1038/srep16379

**Published:** 2015-11-06

**Authors:** Yu Zhou, Yan-Zhen Zheng, Hai-Yuan Sun, Geng Deng, Zhi-Wu Yu

**Affiliations:** 1 Key Laboratory of Bioorganic Phosphorous Chemistry and Chemical Biology (Ministry of Education), Department of Chemistry, Tsinghua University, Beijing 100084, P. R. China

## Abstract

Characterization/identification of the clusters/associates in liquids has long been a challenging topic. In this paper, we report a method to identify molecules with two different existing forms in a binary liquid solution. In this so-called two-state situation, the excess infrared spectra of a vibration mode of the respective molecule will show identical band shape if the other component is transparent in the region. More conveniently, the positions of the positive peak, negative peak, and zero-value will be seen to be fixed with varying compositions of the binary system. In the case of non-two-state mixtures, for example the mere solvation of solute by solvent, those positions will be variable. The conclusions are supported/demonstrated by computational simulation and experiments on two binary systems, D_2_O−H_2_O and C_6_F_5_I−*cyclo*-C_6_H_12_.

Liquids are much more complicated than gases and solids in the view point of theoretical treatment. They do not like gases which are typified by far molecular-separation and thus can be dealt with modified perfect gas law, nor do they like crystals which have definite structures and thus can be treated by the laws of solid-state physics. The biggest challenge in the study of liquids has been to describe their structures^1^.

The earliest models take liquids as totally disordered structures. Later on, it was found that liquids are long range-disordered and short range-ordered^2^. The presence of particular interactions such as hydrogen bonding^3^,^4^, halogen bonding^5^, and π–π stacking interactions^6^, will result in molecular association. It has been hypothesized that the associates could be dimer, trimer, tetramer, and multimers, either in linear, cyclic, or even branched forms^7^,^8^. If the associates are very stable and thus can be considered as discrete species, mass spectroscopy and methods to determine molecular weight such as freezing point depression can be of help. Unfortunately, most associates are not very stable, and can even be short-lived. To identify these not-very-stable associates, therefore, has been a long standing unsolved problem. In this paper, we report our effort to identify the associates using excess infrared spectroscopy.

Infrared spectra provide rich information of molecular structures. The absorption bands, however, are heavily overlapped for liquid samples. The concept of excess spectroscopy, proposed in our laboratory^9^,^10^, can improve the resolution to some extent. For example, by examining the O−H stretching band of tert-butanol–tetrachloride binary mixtures, we were able to identify four species in the mixtures, namely monomer, dimer, trimer, and multimers^10^. Other than this binary system, a number of binary systems including ionic liquids and molecular solvents have been investigated^11^,^12^,^13^. One interesting phenomenon is that the positions of some excess peaks are independent of concentration. As exemplified in Fig. 1(a,b), the excess peaks over the *ν*(O−H) in C_2_H_5_OH−CD_3_CN system (a) and *ν*(O−H) stretching in tert-butanol−CCl_4_ system (b) show fixed positions^10^,^13^.

It should be stressed that not all the excess peaks behave like this. The positions can be concentration-dependent as shown in Fig. 1(c) for *ν*(C ≡ N) stretching in CD_3_CN−CCl_4_ system. Intuitively, the fixed position of an excess band should mean something, most likely a reflection of identical configuration of the concerned bond, plus the same or close solvent environment to the respective vibrational mode. In this paper, we will show that this is true under certain conditions, particularly when there are only two existing forms of the concerned molecules. By changing the concentration of the mixtures, the amount of the two forms will change. This is regarded as a two-state transformation.

The significance of the present work lies also on its correlation to the *in-situ* identification of molecular clusters in liquids. Up to date, there have been some experimental methods to investigate molecular clusters^14^. But they are almost all for gas phase identification. For liquid state samples, for example water, their clusters have been studied by spraying the samples into inert solvent such as liquid helium^15^,^16^. Molecular clusters identified by these methods cannot reflect the real situations in liquids. Recently, Ben-Amotz and co-workers proposed that solvent molecules in a solution can be classified as bulk and solvation-shell molecules^17^,^18^,^19^. They used a multivariate curve fitting method to extract the spectrum of the latter. In principle, the idea can be used to extract the spectra of molecular clusters, while the detailed information of those clusters, including their types and spectroscopic properties, is required to be known. As a matter of fact, we have to accept that unambiguous *in-situ* identification of molecular clusters in pure liquids and liquid mixtures is an unsolved problem. The method proposed in this work gives a solution to the case when there are two existing forms of the target molecule in a binary system. The two forms can be two types molecular clusters or one type cluster plus monomer of the concerned component.

## Results and Discussion

### Theory

We consider a binary system. For each of the two components, the absorbance at a selected wavenumber is described in the form of Beer-Lambert Law:


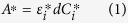


where ε_i_* and *C*_i_* are the molar absorption coefficient and the molarity of the compound i in pure form, redspectively, *d* the light path. In the case of the binary mixtures, we assume the form of Beer-Lambert Law is still applicable:


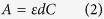


where *C *= *C*_1_+*C*_2_. Generally speaking, absorption coefficient ε of the mixture is not a constant, but a function of wavelength and concentrations.

We define the absorption coefficient of an ideal mixture as:


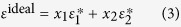


where *x*_1_ and *x*_2_ are mole fractions of the two components. Then, the excess infrared spectrum, in the form of excess absorption coefficient, is defined as follows:


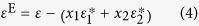


Now we purposely identify an absorption band of component 2 (solute, M), which is not overlapped with absorption bands from the other component (solvent, S). This means that the respective wavenumber range is transparent to the solvent. We assume that there are two existing forms of the solute, for example with and without forming complex with solvent (M_1_ and M_2_), over the entire or part of the concentration range. Their molar absorptivities are denoted as ε_21_ and ε_22_, which are independent on concentrations. The amounts of the two forms of the solute, however, are variable. The fraction of form 1 is denoted as δ over the two forms of the solute. In the absence of solvent, the fraction is δ_0_. With these parameters, Equation (3) turns to be


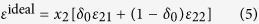


Molar absorptivity of the real binary mixture is,


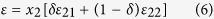


Then, based on Equation (4), excess molar absorptivity of a real binary mixture is,


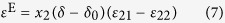


It is thus clear that the term 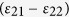 defines the shape of an excess spectrum band, which is independent of the apparent concentration of the binary system. Only when the molar absorptivities of the two forms of the solute equal to each other, the excess function can be zero. Thus we conclude that the peak positions and zero-intensity frequency of the excess spectroscopic bands are all fixed in a series of binary mixtures, if there are only two existing forms of the solute and the solvent is transparent over the examined wavenumber range.

### Digital simulation

With the above theoretical analysis, we have performed some digital simulation of excess infrared spectra to show the ideal appearance of the excess infrared spectra. Here, a single absorption band is expressed by the Lorentzian function as follows,


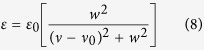


where *w* represents the full width at half-height, *ν*_0_ the center position of band, and ε_0_, the molar absorptivity when ν = *ν*_0_. When there are two forms of the solute molecules in the binary system, the spectral function is expressed as follows,


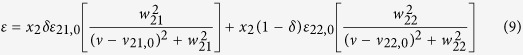


**Figure 2:**
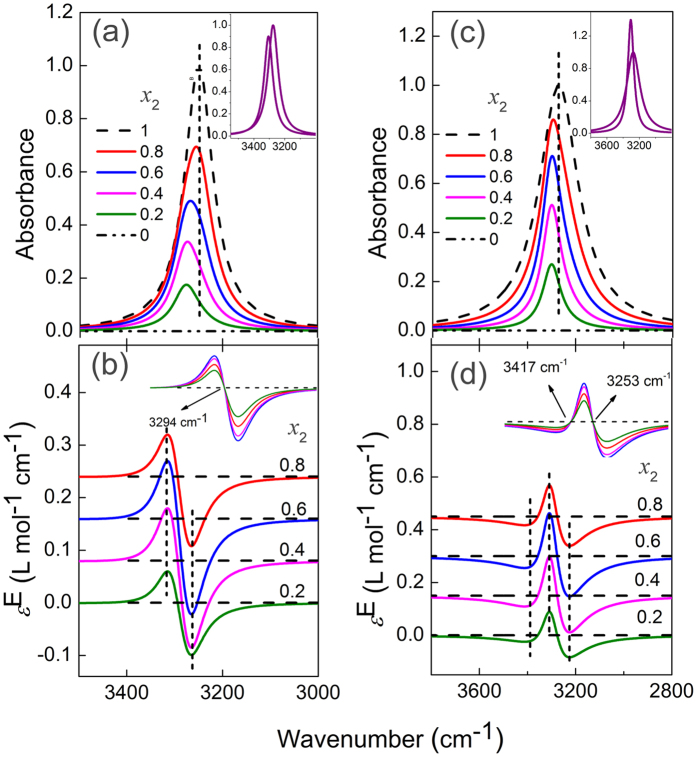
Simulated infrared spectra (**a,c**) and the respective excess spectra (**b,d**) for the two-state transformation system. The dashed and dash-dot dotted lines in (**a,c**) depict spectra of pure M and S. The inserts are: the absorption bands representing the two existing forms of the molecules in their pure states (**a,c**), overlapping presentation of the excess curves (**b,d**).

The two peak positions are arbitrarily set at *ν*_21,0_ = 3275 cm^−1^ (State 1) and *ν*_22,0_ = 3305 cm^−1^ (State 2). By assigning the following parameters: ε_21,0_ = 1, ε_22,0_ = 0.9, *w*_21_ = 80, *w*_22_ = 70, the absorption bands representing the two states of the molecules can be derived and the results are shown in the inset of Fig. 2(a). Assuming pure M only exists in the form of State 1, and when the mole fraction of M decreases, the fraction of State 1 in M also decreases in the M−S binary system. Thus giving a series of apparent concentration *x*_2_ =1, 0.8, 0.6, 0.4, 0.2, and 0, we assume *δ* = 1, 0.7, 0.45, 0.25, 0.1 and 0. IR spectra and excess infrared spectra are simulated and the results are shown in Fig. 2. As can be seen in Fig. 2(a), the absorption peak looks like singular and it shifts gradually to higher wavenumber with increasing solvent concentration. Apparently, the blue shift would have been caused by solvation effect.

The excess infrared spectra shown in Fig. 2(b) provide correct insights of the system. Apart from showing a positive peak at the higher wavenumber side and a negative peak at the lower wavenumber side in each curve, which is the same with solvation effect, the spectra give fixed positions of positive and negative peaks at 3314 and 3264 cm^−1^, respectively. Meanwhile, the zero-value points of the four excess spectra appear at identical position. Different values of the parameters generate excess infrared spectra with different shapes. When there is significant difference between the two *w* values, namely one broad peak overlaps with a very narrow peak, two zero-value points can be seen. Shown in Fig. 2(c,d) is one such example where ε_21,0_ = 1, ε_22,0_ = 1.4, *w*_21_ = 200, *w*_22_ = 90. Even in this case, the positive and negative peak positions and the zero-value positions are all fixed.

We also examined the solvation effect, where an infrared absorption band of component 2 shifts gradually. To do the simulation, we first constructed an absorption peak of component 2 centered at 3275 cm^−1^ with ε_2,0_ = 1 and half peak width *w*_2_ = 80. Then the mole fraction of the component 2 decreases by 0.2 each step, and at the same time the absorption peak shifts to higher wavenumber by 10 cm^−1^. The Lorentzian peak is described as follows


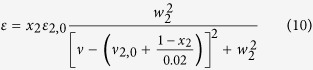


**Figure 3:**
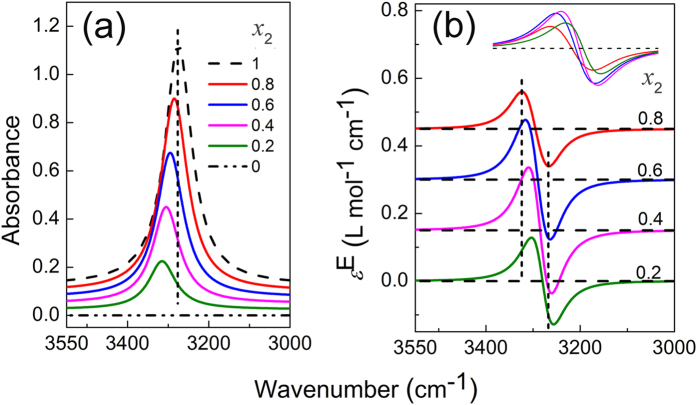
Simulated infrared spectra (**a**) and excess infrared spectra (**b**) in the presence of solvation effect. The dashed and dash-dot dotted lines in (**a**) depict spectra of pure M and S, respectively. The insert is the overlapping presentation of the excess curves.

The simulated infrared spectra as shown in Fig. 3(a) are quite similar to those in Fig. 2(a). The simulated excess infrared spectra, however, show completely different results. The positions of both positive and negative peaks, as well as the position of the zero-value point, are not fixed.

If two distinct states and solvent effects are combined, or there are more than two distinct states of the solute, the Equation (7) will not hold anymore. More or less, the positions of the positive and negative peaks will subject to changes. Nevertheless, our simulation indicates that, depending on the extent of hydration or the relative separation of the multi-state peaks, positions of part or all of the peaks in the excess spectra can be relatively fixed (see Supplementary Information). We, therefore, conclude that if the position of an excess peak is relatively fixed, most likely it represents a distinct species.

### D_2_O**−**H_2_O system

The hydrogen/deuterium atoms in a water molecule are not bonded to one molecule forever, but rather they can exchange with the hydrogen/deuterium in the neighboring molecules. Consequently, the H and D atoms in D_2_O−H_2_O mixture will switch one another to form the third species, HDO^20^,^21^. In D_2_O−H_2_O binary system, there are two kinds of O−D, those in HDO and in D_2_O. This is the case of two-state transformation upon changing concentration.

**Figure 4:**
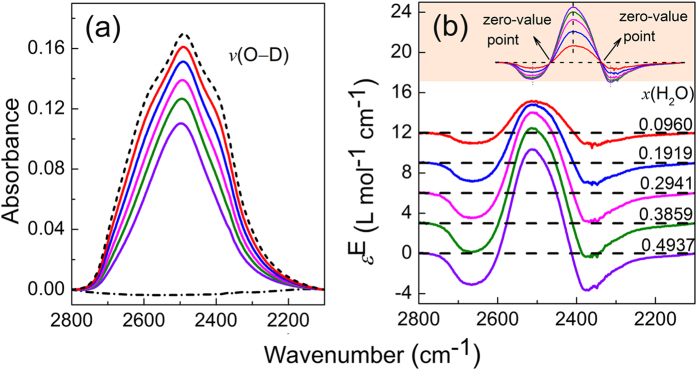
Infrared spectra (**a**) and the respective excess infrared spectra (**b**) of D_2_O–H_2_O system in the range of the O–D stretching vibration. The dashed and dash-dot dotted lines in (**a**) depict spectra of pure D_2_O and H_2_O, respectively.

The partial ATR-FTIR and excess spectra of D_2_O−H_2_O system in the O−D stretching region are shown in Fig. 4. Normal water has no absorptions in the wavenumber region. The absorption band in Fig. 4(a) is assigned to ν(O−D) according to literature^20^. In Fig. 4(b), the excess spectra show two fixed-position negative peaks, one fixed positive peak, and two fixed zero-value points, which are the same as the simulation result shown in Fig. 2(d). This demonstrates that, in the binary system with two-state transformation, both the excess peaks and zero-value points are fixed.

### C_6_F_5_I**−** *cyclo*-C_6_H_12_ system

**Figure 5:**
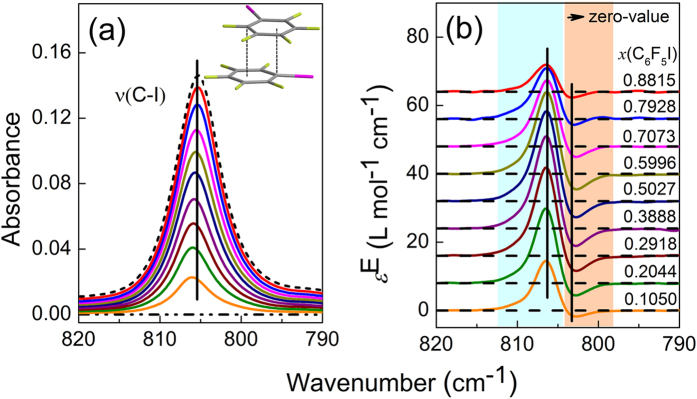
Infrared spectra (**a**) and excess infrared spectra (**b**) of the C_6_F_5_I–*cyclo*-C_6_H_12_ system at different mole fraction of C_6_F_5_I in the wavenumber range of the C–I stretching vibration. The dashed and dash-dot dotted lines in (**a**) depict spectra of pure C_6_F_5_I and *cyclo*-C_6_H_12_, respectively.

The C_6_F_5_I–*cyclo*-C_6_H_12_ system was also studied by ATR-FTIR technique. The IR and excess infrared spectra of the C_6_F_5_I–*cyclo*-C_6_H_12_ system in the C–I stretching region are shown in Fig. 5. The band at 805 cm^−1^ is attributed to the C−I stretching vibration^22^. In Fig. 5(a), from top to bottom, the peak position is blue-shifted. In Fig. 5(b), the excess spectra show fixed positions of positive peak, negative peak, and zero-value points, which are in consistent with the simulation results shown in Fig. 2(b). This strongly suggests that C_6_F_5_I–*cyclo*-C_6_H_12_ system exists in two distinct states upon dilution with *cyclo*-C_6_H_12_. For C_6_F_5_I, *cyclo*-C_6_H_12_ is an inert solvent. So the two states may be attributed to the absorptions of monomer and self-aggregated C_6_F_5_I^12^. Most likely, the self-aggregated C_6_F_5_I is the dimer of the molecule. Quantum chemical calculations were performed to find the optimized structure of the C_6_F_5_I dimer, which is shown in the inset of Fig. 5(a). As can be seen, the two molecules form a displaced π−π stacking interaction pair. The distance of the two planes is 0.303 nm. The interaction energy is −100.25 kJ/mol, suggesting the dimer is quite stable. Furthermore, the calculated frequencies of *v*(C−I) indicate that there is a redshift upon formation of dimer. Thus the negative peak at lower wavenumber is assigned to dimer and the positive one to monomer.

## Conclusions

In this paper, we developed a method to differentiate two-state and non-two-state of molecular exiting forms in binary liquid mixtures, based on the excess spectroscopic technique. If a molecule exists in two forms over a range of concentration in a binary mixture, the excess band of a vibration mode of the molecule will show fixed peak positions and zero-intensity frequencies in the concentration range if the other component has no absorption in the region. Otherwise, these positions should be concentration-dependent. These features are demonstrated with numerical simulation and experiments. In D_2_O−H_2_O system, there are two O−D stretching forms. They are readily assigned to that from D_2_O and DOH. In C_6_F_5_I–*cyclo*-C_6_H_12_ system, the excess infrared spectra in the C−I stretching region suggest that there are two forms of C_6_F_5_I during the mixing process. They are most likely self-aggregated and non-aggregated C_6_F_5_I. The aggregation is believed to be in the form of displaced π−π stacking. For more complicated situations such as the coexistence of three or more forms of the concerned component, the feature/shape of the excess spectra will be concentration-dependent. But if the position of a positive peak or negative peak in a series of excess spectra is relatively fixed, most likely it indicates a distinct species.

## Methods

### Chemicals

D_2_O (>99%) was purchased from Armar Chemicals. C_6_F_5_I (>98%) was purchased from J&K Scientific. *Cyclo*-C_6_H_12_ (>99%) was from Beijing Chemical Plant (Beijing, China). The samples were used without further purification.

### Sample preparation

A series of D_2_O−H_2_O and C_6_F_5_I–*cyclo*-C_6_H_12_ binary mixtures were prepared by weighing. The mole fractions of H_2_O in D_2_O−H_2_O mixtures are 1, 0.4937, 0.3859, 0.2941, 0.1919, 0.0960, and 0. The mole fractions of C_6_F_5_I in C_6_F_5_I–*cyclo*-C_6_H_12_ mixtures are 1, 0.8815, 0.7928, 0.7073, 0.5996, 0.5027, 0.3888, 0.2918, 0.2044, 0.1050, and 0.

### FTIR spectroscopy

FTIR spectra over the range from 4000 to 650 cm^−1^ were collected at room temperature (~25 °C) using a Nicolet 5700 FTIR spectrometer, equipped with an MCT detector. Two ATR cells made of trapezoidal ZnSe/Ge crystals were used in the experiments. A ZnSe crystal with an incident angle of 45° and 12 reflections was used to examine the stretching bands of O−D in D_2_O−H_2_O system. A Ge crystal with an angle of 60° and 7 reflections was used to examine the strong stretching bands of C−I in C_6_F_5_I–*cyclo*-C_6_H_12_ system. Spectra were recorded with a resolution of 2 cm^−1^, a zero filling factor of 2, and 32 parallel scans. The refractive indexes of solutions were measured with a refractometer at 25 °C. The formulas suggested by Hansen^23^, were used to do the ATR corrections.

### Excess infrared spectroscopy

The theory of excess infrared spectroscopy has been described in detail elsewhere^9^,^10^. Briefly, an excess infrared spectrum is defined as the difference between the spectrum of a real solution and that of the respective ideal solution under identical conditions. The working equation in calculating the excess infrared spectrum is as follows:


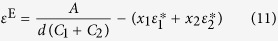


where *A* is the absorbance of the mixture, *d* is the light path length, *C*_1_ and *C*_2_ are molarities of the two components, *x*_1_ and *x*_2_ are mole fractions of components 1 and 2, and *ε*_1_^*^ and *ε*_2_^*^ are molar absorption coefficients of the two components in their pure states, respectively. The calculation of the excess infrared spectra was programmed using Matlab 7.0 (Math Works Inc., Natick, MA).

### Quantum chemical calculations

Quantum chemical calculations were performed by employing second-order many-body perturbation theory (MP2) at mixed basis sets with the Gaussian 09 program^24^. The molecular geometry were fully optimized with the LANL2DZ^25^, basis set for iodine atom and the aug-cc-pVDZ^26^, basis set for hydrogen, carbon, and fluorine atoms. All the optimized geometries were recognized as local minima with no imaginary frequency.

## Additional Information

**How to cite this article**: Zhou, Y. *et al.* Two-State or Non-Two-State? An Excess Spectroscopy-based Approach to Differentiate the Existing Forms of Molecules in Liquids Mixtures. *Sci. Rep.* **5**, 16379; doi: 10.1038/srep16379 (2015).

## Supplementary Material

Supplementary Information

## Figures and Tables

**Figure 1:**
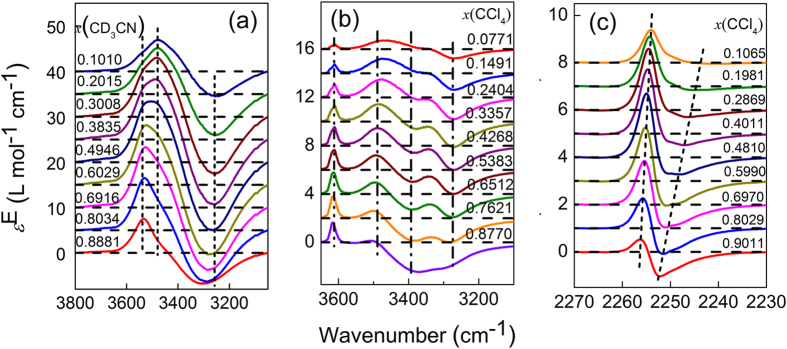
Excess infrared spectra. (**a**) O–H stretching in C_2_H_5_OH−CD_3_CN system^13^. (**b**) O–H stretching in tert-butanol–CCl_4_ system^10^. (**c**) C ≡ N stretching in CD_3_CN–CCl_4_ system. Mole fractions of the binary mixtures have been indicated by the respective curves in the figure.

## References

[b1] Reichardt C. & Welton T. Solvents and Solvent Effects in Organic Chemistry 4th edn. Ch. 1, 1–6 (John Wiley & Sons, 2011).

[b2] Ise N. & Okubo T. Ordered distribution of electrically charged solutes in dilute solutions. Acc. Chem. Res. 13, 303–309 (1980).

[b3] Gadre S. R., Yeole S. D. & Sahu N. Quantum chemical investigations on molecular clusters. Chem. Rev. 114, 12132–12173 (2014). 25341561 10.1021/cr4006632

[b4] Guo J. H. *et al.* Molecular structure of alcohol-water mixtures. Phys. Rev. Lett. 91, 157401 (2003). 14611492 10.1103/PhysRevLett.91.157401

[b5] Metrangolo P., Neukirch H., Pilati T. & Resnati G. Halogen bonding based recognition processes: A world parallel to hydrogen bonding. Acc. Chem. Res. 38, 386–395 (2005). 15895976 10.1021/ar0400995

[b6] Sharma B., Srivastava H. K., Gayatri G. & Sastry G. N. Energy decomposition analysis of cation–π, metal ion–lone pair, hydrogen bonded, charge-assisted hydrogen-bonded, and π–π interactions. J. Comput. Chem. 36, 529–538 (2015). 25581071 10.1002/jcc.23827

[b7] Vaitheeswaran S., Yin H., Rasaiah J. C. & Hummer G. Water clusters in nonpolar cavities. Proc. Natl. Acad. Sci. USA 101, 17002–17005 (2004). 15572444 10.1073/pnas.0407968101 PMC535395

[b8] Saykally R. J. & Blake G. A. Molecular interactions and hydrogen bond tunneling dynamics: some new perspectives. Science 259, 1570–1575 (1993). 17733020 10.1126/science.259.5101.1570

[b9] Li Q. Z., Wu G. S. & Yu Z. W. The role of methyl groups in the formation of hydrogen bond in DMSO-methanol mixtures J. Am. Chem. Soc. 128, 1438–1439 (2006). 16448100 10.1021/ja0569149

[b10] Li Q. Z., Wang N. N., Zhou Q., Sun S. Q. & Yu Z. W. Excess infrared absorption spectroscopy and its applications in the studies of hydrogen bonds in alcohol-containing binary mixtures. Appl. Spectrosc. 62, 166–170 (2008). 18284791 10.1366/000370208783575663

[b11] Zhang Q. G., Wang N. N. & Yu Z. W. Hydrogen bonding interactions between the ionic liquid 1-ethyl-3-methylimidazolium ethyl sulfate and water. J. Phys. Chem. B 114, 4747–4754 (2010). 20337406 10.1021/jp1009498

[b12] Zheng Y. Z., Deng G., Zhou Y., Sun H. Y. & Yu Z. W. A Comparative study of halogen-bond and hydrogen-bond interactions between benzene derivatives and dimethyl sulphoxide. Chem Phys Chem 16, 2594–2601 (2015). 26118800 10.1002/cphc.201500324

[b13] Zhou Y., Zheng Y. Z., Sun H. Y., Deng G. & Yu Z. W. Hydrogen bonding interactions in ethanol and acetonitrile binary system: A near and mid-infrared spectroscopic study. J. Mol. Struct. 1069, 251–257 (2014).

[b14] Ludwig R. Water: From clusters to the bulk. Angew. Chem. Int. Ed. 40, 1808–1827 (2001) 11385651

[b15] Nauta K. & Miller R. E. Formation of cyclic water hexamer in liquid helium: The smallest piece of ice. Science 287, 293–295 (2000). 10634781 10.1126/science.287.5451.293

[b16] Mir M. H. & Vittal J. J. Phase transition accompanied by transformation of an elusive discrete cyclic water heptamer to a bicyclic (H_2_O)_7_ cluster. Angew. Chem. Int. Ed. 46, 5925–5928 (2007). 10.1002/anie.200701779 17577896

[b17] Fega K. R., Wilcox D. S. & Ben-Amotz D. Application of Raman multivariate curve resolution to solvation-shell spectroscopy. Appl. Spectrosc. 66, 282–288 (2012). 22449304 10.1366/11-06442

[b18] Perera P., Wyche M., Loethen Y. & Ben-Amotz D. Solute-induced perturbations of solvent-shell molecules observed using multivariate Raman curve resolution. J. Am. Chem. Soc. 130, 4576–4577 (2008). 18336023 10.1021/ja077333h

[b19] Davis J. G., Gierszal K. P., Wang P. & Ben-Amotz D. Water structural transformation at molecular hydrophobic interfaces. Nature 491, 582–585 (2012). 23172216 10.1038/nature11570

[b20] Maxa J. J. & Chapadosb C. Isotope effects in liquid water by infrared spectroscopy. J. Chem. Phys. 116, 4626–4642 (2002).

[b21] Saihara K., Yoshimura Y., Ohta S. & Shimizu A. Properties of water confined in ionic liquids. Sci. Rep. 5, 10619 (2015). 26024339 10.1038/srep10619 PMC4448549

[b22] Frankiss S. & Harrison D. Thermodynamic properties of fluorine compounds—XVI. The vibrational spectra and thermodynamic functions of pentafluorobenzene, chloropentafluorobenzene, bromopentafluorobenzene and methylpentafluorobenzene. Spectrochimica Acta Part A: Mol. Spectrosc. 31, 1839–1864 (1975).

[b23] Hansen W. N. Expanded Formulas for Attenuated Total Reflection and the Derivation of Absorption Rules for Single and Multiple ATR Spectrometer Cells. Spectrochim. Acta 21, 815–833 (1965).

[b24] Frisch M. J. *et al.* Gaussian 09, Gaussian, Inc.: Wallingford, CT, 2009.

[b25] Wadt W. R. & Hay P. J. Ab initio effective core potentials for molecular calculations. Potentials for the transition metal atoms Sc to Hg. J. Chem. Phys. 82, 270 (1985).

[b26] Dunning T. H. Gaussian basis sets for use in correlated molecular calculations. I. The atoms boron through neon and hydrogen. J. Chem. Phys. 90, 1007 (1989).

