# Supplementary material for: Two-State or Non-Two-State? An Excess Spectroscopy-based Approach to Differentiate the Existing Forms of Molecules in Liquids Mixtures
Source: Sci Rep. 2015 Nov 6;5:16379. doi: 10.1038/srep16379 (PMC4635405; doi:10.1038/srep16379)
Supplement: Supplementary Information [file srep16379-s1.pdf]

Supplementary Information for

Two-State or Non-two States? An Excess Spectroscopy-based Approach to Differentiate the Existing Forms of Molecules in Liquids Mixtures

Yu Zhou, Yan-Zhen Zheng, Hai-Yuan Sun, Geng Deng & Zhi-Wu Yu*

Key Laboratory of Bioorganic Phosphorous Chemistry and Chemical Biology (Ministry of Education), Department of Chemistry, Tsinghua University, Beijing 100084, P. R. China

Simulation on the combination of solvation effect and presence of two existing forms of solute.

Solvation effect is the result of weak non-specific interactions. This is different from the presence of two distinct states of the solute, which is resulted from strong specific molecular interactions. We have performed simulations using the same parameters as in Figure 2a&b, except a blue shift of 2 cm^{-1} when the mole fraction of M decreases by 0.2. The Lorentzian peak is described as follows:

$$\varepsilon = x_2\delta\varepsilon_{21,0}\frac{w_{21}^2}{\left[\nu-\left(\nu_{2,0}+\frac{1-x_2}{0.02}\right)\right]^2+w_{21}^2}+x_2\left(1-\delta\right)\varepsilon_{22,0}\frac{w_{22}^2}{\left[\nu-\left(\nu_{2,0}+\frac{1-x_2}{0.02}\right)\right]^2+w_{22}^2}$$

The derived IR spectra and the respective excess spectra are shown in Figure S1. Clearly, different from the significant blue shift shown in (a), with decreasing mole fraction, the positive, negative, and zero-value positions of the four excess spectra are almost fixed.

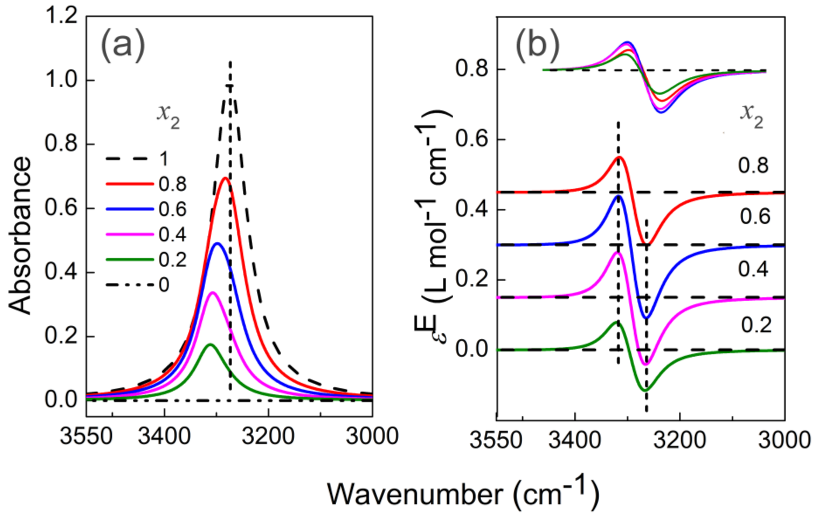

Figure S1. Simulated infrared spectra (a) and the respective excess infrared spectra (b) for the combination of two states and solvation effects. The dashed and dash-dot dotted lines in (a) depict spectra of pure M and S. The insert is the overlapping presentation of the excess curves.
